# Assessment of the ability of HPV vaccines to induce neutralizing antibodies based on pseudovirus-based neutralization assay

**DOI:** 10.3389/fpubh.2025.1636491

**Published:** 2025-09-01

**Authors:** Zhihua Wu, Xuejie Li, Beibei Zhang

**Affiliations:** ^1^Department of Clinical Laboratory, Tongde Hospital of Zhejiang Province, Hangzhou, China; ^2^The First Affiliated Hospital of Zhejiang Chinese Medical University (Zhejiang Provincial Hospital of Chinese Medicine), Hangzhou, China; ^3^Huzhou Central Blood Station, Huzhou, China

**Keywords:** Human papillomavirus (HPV), HPV vaccines, neutralizing antibodies, pseudovirus-based neutralization assay (PBNA), neutralization titer

## Abstract

**Introduction:**

Human papillomavirus (HPV) infection represents a significant public health concern due to its strong association with various cancers and other diseases. The development and implementation of prophylactic HPV vaccines, including bivalent, quadrivalent, and nonavalent formulations, have markedly reduced the incidence of these diseases by inducing the production of neutralizing antibodies. The detection and characterization of neutralizing antibodies against HPV are essential for understanding immune responses and evaluating vaccine efficacy. However, limited data are available on neutralizing antibody levels following vaccination with different HPV vaccines in the Chinese population. This study aimed to evaluate neutralizing antibody responses in individuals vaccinated with HPV vaccines.

**Methods:**

Eighty-four female vaccine recipients were enrolled. Neutralizing antibody responses were assessed using a pseudovirus-based neutralization assay (PBNA). Serum samples were analyzed for the presence and titers of neutralizing antibodies against HPV types 6, 11, 16, 18, 31, 33, 45, 52, and 58.

**Results:**

The study revealed variations in neutralizing antibody levels across different HPV subtypes. The PBNA demonstrated high reproducibility and reliability in measuring these responses.

**Discussion:**

These findings highlight the utility of PBNA as a robust tool for assessing immune responses to HPV vaccination and optimizing vaccine strategies. The study provides valuable insights into the neutralizing antibody responses elicited by HPV vaccines in the Chinese population.

## 1 Introduction

Human papillomavirus (HPV) is a highly prevalent sexually transmitted pathogen, with an estimated 75 to 80% of individuals worldwide acquiring the virus at some point in their lives (1). Persistent HPV infection is strongly associated with the development of various cancers, including cervical, anal, and oropharyngeal cancers (2). Of the more than 200 identified HPV genotypes, 15 are classified as high-risk types (HPV16, 18, 31, 33, 35, 39, 45, 51, 52, 56, 58, 59, 68, 73, and 82) (3, 4). Notably, at least 98% of cervical cancers are attributed to persistent infection with these high-risk HPV genotypes.

HPV infection is typically asymptomatic in immunocompetent individuals but can reactivate in those with compromised immune systems. The relationship between HPV pathogenesis and the host immune response is complex (5). The innate immune response serves as the first line of defense against HPV infection, with the secretion of various cytokines playing a critical role in identifying and responding to HPV-infected epithelial cells (6). During the adaptive immune response, multiple immune cells are involved in combating HPV infection (7). However, HPV can impair the adaptive immune response by targeting CD4 + and CD8 + T lymphocytes, effectively disrupting immune surveillance (8–10). Humoral immunity against natural HPV infection is primarily directed against epitopes in the L1 protein, which is the main structural component of the viral capsid (11). The pseudovirus-based neutralization assay (PBNA) is widely regarded as the “gold standard” for detecting neutralizing antibodies because it measures functional antibody activity rather merely binding capacity (12, 13).

Prophylactic vaccination has been shown to be an effective strategy for preventing HPV-related diseases by activating humoral immunity and inducing the production of virus-neutralizing antibodies, which provide protection against HPV infection (14). Despite this, according to a 2021 survey, only 3% of all Chinese females, and just 1.9% of those aged 9 to 14 years, had received the HPV vaccine (15). In 2018, following extensive clinical research, the U.S. Food and Drug Administration (FDA) expanded the approved age range for the nonavalent HPV vaccine to include individuals aged 27 to 45 years (16, 17). Neutralizing antibodies play a critical role in preventing HPV infection by specifically targeting and neutralizing infectious HPV particles, thereby blocking their entry into host cells (18). Assessing the levels of neutralizing antibodies in vaccinated individuals is essential for evaluating vaccine efficacy and monitoring immune responses. The aim of this study was to investigate the neutralizing antibody responses against HPV types 6, 11, 16, 18, 31, 33, 45, 52, and 58 in a cohort of vaccinated individuals. Specifically, this study sought to analyze neutralizing antibody levels at different time points post-vaccination, compare the ability of different HPV vaccine types to induce neutralizing antibodies, and assess differences in neutralizing antibody levels induced by allogeneic vaccines across different age groups.

## 2 Materials

### 2.1 Sample collection and processing

- The number of recipients of nonavalent, quadrivalent and bivalent HPV vaccines was 52, 22 and 10, respectively, for total of 84 participants after excluding 6 participants with biologically implausible 12-month bivalent vaccine data. Recipient age statistics for each group are shown in the table, and there was no statistical difference in age between the groups.
- Study participants were required to have completed the full three-dose vaccination series according to manufacture recommendations. Dosing schedules were: bivalent vaccine (0, 1, 6 months), quadrivalent vaccine (0, 2, 6 months), and nonavalent vaccine (0, 2, 6 months). Participants with incomplete vaccination or significant schedule deviations (>4 weeks from recommended intervals) were excluded from analysis.
- Serum samples for neutralizing antibody assessment were collected at multiple timepoints after completion of the vaccination (1, 6, 12 months) depending on participant follow-up schedules.
- Serum isolation: 3,000 rpm × 5 min.
- Storage: −80°C long term.

To ensure data integrity, all specimens were randomly coded and processed under blinded conditions throughout the laboratory procedures.

HPV DNA screening was not performed at study enrollment. Participants were not excluded based on prior HPV infection history, which represents a limitation of the current study design.

### 2.2 Human embryonic kidney 293FT cells

Human embryonic kidney 293FT cells were purchased from Zhejiang Meisen Cell Technology Company. (Item number: CTCC-001-0046. Catalog Number: R700-07).

### 2.3 HPV pseudovirus

- HPV type 6, 16 and 31 pseudoviral particles consisted of L1 and L2 proteins and a GFP reporter gene.
- HPV types 11, 18 and 33 pseudoviral particles consisted of L1 and L2 proteins and an RFP reporter gene.
- HPV types 45, 52, and 58 pseudoviral particles consist of L1 and L2 proteins and an E2FP reporter gene.

### 2.4 DMEM medium preparation

The DMEM medium was prepared by combining 10% fetal bovine serum, 1% penicillin–streptomycin double antibody, and 89% DMEM high-glucose medium.

## 3 PBNA method experimental principle

PBNA was selected as the primary methodology due to its superior ability to detect functional neutralizing antibodies and provide type-specific, quantitative measurements independent of vaccine formulation.

The L1 and L2 proteins, along with reporter genes, self-assemble into HPV pseudovirus particles. Neutralizing antibodies can neutralize the pseudovirus *in vitro*, thereby preventing it from infecting host cells. If the pseudovirus successfully enters the cell, it will express fluorescent proteins, which can be detected and quantified using appropriate instruments. By comparing the fluorescence intensity to that of the control group, the dilution at which 50% of the pseudovirus is inhibited by the antibodies (ID50) can be calculated.

## 4 Detailed protocol for PBNA in HPV vaccine studies

### 4.1 Pseudovirus titration

Seed 293FT cells into two 96-well cell culture plates with a density of 1.5 × 10^4^ cells/100 μl per well. Allow the cells to adhere for 6 h and incubate at 37°C in 5% CO2 incubator.

Dilute pseudoviruses 10^6^,10^7^,10^8^, and 10^9^ fold to a final volume of 1 ml.

Add 100 μl of the diluted pseudovirus to each well of a 96-well culture plat, ensuring eight replicate wells of each dilution.

Incubate the cell culture plates at 37°C in 5% CO2 incubator for 72 h.

Observe and record the number of wells with positive fluorescent cells at each dilution using a fluorescence microscope.

Calculate the tissue culture infectious does 50% (TCID50), defined as the viral dilution at which 50% of the cells are infected, using the Reed-Muench method (19, 20).

For each viral dilution, record the number of positive (a) and negative (b) wells.

Calculate the cumulative number of positive and negative wells:

- Accumulate the positive wells from the bottom up (c)
- Accumulate the negative wells from the top down (d);

Calculate the percentage of positive wells using the formula:


Ratio(%)=[c/(c+d)]×100

Calculate the distance ratio using the formula:


$$\begin{array}{l} \text{Distance Ratio}=(\text{Percentage of lesions above}\;50–50\%)\\ /(\text{Percentage of lesions above}\;50\%-\text{Percentage of lesions below}\;50\%) \end{array}$$

Calculate lgTCID50 using the formula: The distance ratio is added to the virus dilution above 50% of the lesion, which is the TCID50 for that virus.

lgTCID50 = Distance Ratio × (Difference between logarithms of dilutions) + (Logarithm of dilutions above 50% lesion rate).

### 4.2 Cell preparation

Pre-seed 293FT cells into a flat-bottom 96-well plate for 4–8 h at 100 μl/ well (1.5 × 10^4^ cells/well). Incubate the plate at 37°C in a 5% CO2 incubator.

### 4.3 Serum sample preparation

Heat-inactivate serum at 56°C for 30–60 min.

### 4.4 Initial serum dilution

Perform 40-fold initial dilution for the serum:

- Add 152 μl of DMEM complete medium to wells B4- B11 of a 96-well U-bottom plate.
- Add 120 μl of DMEM complete medium to the remaining wells.
- Add 8 μl of the serum sample to each well in B4-B11.

### 4.5 Serial dilution of serum samples

Using a multichannel pipette, adjust pipetting to 40 μl and mixing to 100 μl. Perform the following steps:

- Gently and repeatedly pipette up and down 8 times in wells B4-B11 to ensure thorough mixing.
- Transfer 40 μl of the mixture to the corresponding wells C4-C11. Mix as before.
- Repeat the process until wells G4-G11 are reached. Discard 40 μl of liquid from wells G4-G11 to maintain equal volumes across all wells.

### 4.6 Pseudovirus dilution

Dilute pseudoviruses with DMEM complete medium according to the manufacturer’s instruction; add 120 μl of the diluted pseudovirus working solution to wells B3-G11.

### 4.7 Incubation of serum-Pseudovirus mixture

Leave the dilution plate at 4°C for 1 h.

### 4.8 Infection setup

Pipette 100 μl of the pseudovirus-serum mixture (or medium) from each well of the dilution plate. Gently add this mixture to the corresponding wells of the cell culture plate, where cells have already adhered. Gently tap the plate to ensure even mixing.

### 4.9 Incubation of cell culture plates

Incubate the cell culture plates at 37°C in a 5% CO₂ incubator for 60–96 h.

### 4.10 Fluorescence detection

Detect and count fluorescence points using the Operetta CLS High Content instrument and the Harmony 4.9 analysis system.

### 4.11 Infection inhibition rate calculation

Calculate the infection inhibition rate using the following formula:


$$\begin{array}{l} \text{Inhibition Rate}\;(\%)=[1-(\begin{array}{l} \text{Sample Detection Value}\\ -\text{Cell Control Value} \end{array})]\\ /(\text{Virus Control Value}-\text{Cell Control Value})\times 100 \end{array}$$

### 4.12 Calculation of ID50

Calculate the half-maximal inhibitory dose (ID50), defined as the reciprocal of the serum dilution that inhibits 50% of pseudovirus infection.

### 4.13 Quality control measures

Added standardization procedures following established PBNA protocols (21) and laboratory quality standards (22).

## 5 Statistical analysis

The ID50 for each sample was calculated by fitting a four-parameter logistic equation to determine the best-fit parameters. Statistical analyses were performed using GraphPad Prism version 9.3.0. Comparisons of samples between two groups were performed using independent t-test or nonparametric Mann–Whitney U test. Comparisons of three and more groups were made using ANOVA or Kruskal-Wallits test. For Table 1 temporal analysis, between-group comparisons at each timepoint were performed using Kruskal-Wallits test due to non-normal distribution of antibody titers (confirmed by Shapiro–Wilk test). Post-hoc pairwise comparisons were conducted using Mann–Whitney U tests. No longitudinal or cross-timepoint statistical comparisons were performed to avoid methodological confounding from mixed timepoint data. Additionally, to address potential confounding factors, multivariate linear regression analyses. The models included vaccine type as the primary variable, with age, BMI, time to sampling (days), allergy history, and health status as covariates. Log-transformed antibody titers were used as dependent variable to meet normality assumptions. Model fit was assessed using R^2^ values and residual analysis. (P <0.05) was considered statistically significant.

**Table 1:** Levels of neutralizing antibodies against HPV16 and HPV18 at 1-, 6-, and 12-month timepoints after vaccination with different valences.

Timepoint	Neutralizing antibody	Nonavalent		Quadrivalent		Bivalent		*p*
		Mean (Median)	GMT	Mean (Median)	GMT	Mean (Median)	GMT	
1-month	Anti-HPV16	13,486 (8,250)	9,545	8,225 (8,150)	8,222	8,705 (8,614)	8,702	0.5082
	Anti-HPV18	7,132 (4,450)	4,583	4,450 (4,450)	4,449	7,715 (7,500)	7,685	0.0896
6-month	Anti-HPV16	4,626 (5,000)	3,580	7,680 (7,750)	7,612	10,129 (8,700)	9,849	<0.0001
	Anti-HPV18	8,737 (9,500)	7,740	4,360 (4,550)	4,267	8,357 (7,200)	8,138	0.0004
12-month	Anti-HPV16	6,539 (7,500)	4,638	6,812 (8,350)	4,758			0.2482
	Anti-HPV18	11,388 (13,000)	7,181	4,010 (4,900)	2,271			0.0006

Statistical comparisons performed between vaccine groups at each timepoint using Kruskal-Wallis test, followed by Mann–Whitney U tests. Sample sizes: 1-month (Nonavalent *n* = 8, Quadrivalent *n* = 4, Bivalent *n* = 3); 6-month (Nonavalent *n* = 20, Quadrivalent *n* = 10, Bivalent *n* = 7); 12-month (Nonavalent *n* = 24, Quadrivalent *n* = 8). *p < 0.05, **p < 0.01, ***p < 0.001, ****p < 0.0001; ns = not significant (*p* > 0.05). Long-term immunogenicity assessment of the bivalent vaccine was limited by small sample size and potential measurement artifacts at the 12-month timepoint, requiring exclusion of these data from comparative.

## 6 Results

Using a standardized pseudovirus-based neutralization assay (PBNA), we quantitatively evaluated type-specific neutralizing antibody responses in 84 vaccine recipients, including 52 recipients of the nonavalent vaccine, 22 recipients of the quadrivalent vaccine, and 10 recipients of the bivalent vaccine. All participants completed the full vaccination series according to recommended schedules, ensuring consistent vaccine exposure for comparative analysis.

### 6.1 Type-specific neutralizing antibody analysis

**Figure 1:**
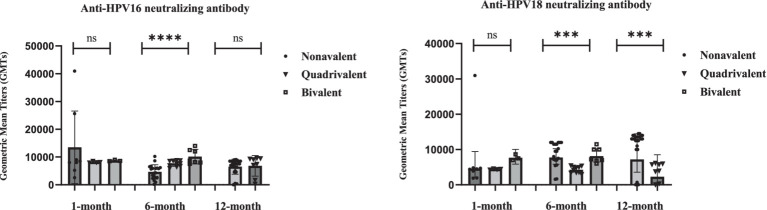
Statistical analysis of neutralizing antibody levels against HPV16 and HPV18 at 1-, 6-, and 12-month after vaccination with different valences. Individual data points are shown as dots. Error bars represents Geometric Mean with 95% CI. Statistical comparisons performed between vaccine groups at each timepoint using Kruskal-Wallis test, followed by pairwise Mann–Whitney U test for significant overall differences. Sample sizes: 1-month (Nonavalent *n* = 8, Quadrivalent *n* = 4, Bivalent *n* = 3); 6-month (Nonavalent *n* = 20, Quadrivalent *n* = 10, Bivalent *n* = 7); 12-month (Nonavalent *n* = 24, Quadrivalent *n* = 8). Longitudinal data with varying sample sizes per timepoint and vaccine type. Individual data points are overlaid on box plots. *p < 0.05, **p < 0.01, ***p < 0.001, ****p < 0.0001; ns = not significant (*p* > 0.05).

Neutralizing antibody responses against HPV16 and HPV18 exhibited distinct kinetic profiles among recipients of the nonavalent, quadrivalent, and bivalent HPV vaccines at the 1-month, 6-month, and 12-month timepoints (Table 1; Figure 1). Due to small sample size and biologically implausible values, 12-month data for the bivalent vaccine group were excluded from analysis. Neutralizing antibody levels against HPV16 and HPV18 1 month after vaccination did not differ significantly between the three vaccine types. The nonavalent vaccine initially induced the highest anti-HPV16 titers (GMT = 9,545 at 1-month) but showed a sharp decline by 6 months (GMT = 3,580).

For HPV18, the nonavalent vaccine exhibited delayed immunogenicity (GMT peak at 6 months), and the quadrivalent vaccine showed progressive waning, with a 49% reduction by 12 months. At the 6 months after vaccination, the bivalent HPV vaccine demonstrated significantly higher neutralizing antibody levels against HPV16 and HPV18 compared to the nonavalent and quadrivalent HPV vaccines (*p* < 0.001, Kruskal-Wallis test). Twelve-month data for the bivalent vaccine were excluded due to small sample size and potential measurement artifacts. These findings reveal fundamentally distinct durability patterns, with the bivalent formulation demonstrating unexpected persistence compared to multivalent vaccines.

### 6.2 Age-stratified immunogenicity analysis for nonavalent vaccine

Given the clinical importance of age-related vaccine responses, we performed a detailed analysis comparing neutralizing antibody levels between different age groups among nonavalent vaccine recipients.

As shown in Table 2, we categorized 52 recipients of the nonavalent HPV vaccine into two age groups and observed that the geometric mean titer (GMT) of neutralizing antibodies was higher in individuals older than 26 years compared to those aged 26 years or younger. A statistically significant disparity in neutralizing antibody titers was observed between the two age cohorts, with the nonavalent HPV vaccine eliciting superior immunogenicity in individuals aged >26 years (*p* < 0.0001).

**Table 2:** Analysis of neutralizing antibody levels in 52 nonavalent HPV vaccine recipients grouped by age.

Neutralizing antibody	≤26 years		>26 years		*p*
	Mean (Median)	GMT	Mean (Median)	GMT	
Anti-HPV6	5,371 (5,500)	4,440	10,096 (8,950)	8,256	<0.0001
Anti-HPV11	4,626 (4,600)	3,576	9,185 (8,300)	6,195	<0.0001
Anti-HPV16	8,643 (9,500)	6,730	14,248 (13,500)	9,804	<0.0001
Anti-HPV18	4,811 (5,100)	3,559	8,837 (84,00)	7,269	<0.0001
Anti-HPV31	2,332 (2,560)	1864	5,561 (4,000)	3,375	<0.0001
Anti-HPV33	26,827 (8,800)	8,414	14,993 (15,500)	13,338	<0.0001
Anti-HPV45	3,099 (3,000)	2,500	4,931 (5,400)	4,365	<0.0001
Anti-HPV52	2,148 (2,300)	1721	3,494 (3,700)	3,398	<0.0001
Anti-HPV58	3,936 (3,300)	2,781	6,033 (6,000)	5,406	<0.0001

Sample sizes: ≤26 years (*n* = 33), >26 years (*n* = 19). Comparisons between age groups performed using Mann–Whitney U test. Age-stratified analysis of neutralizing antibodies measured at mixed timepoints after vaccination series completion.

The consistently higher antibody responses in the >26 years group across all HPV types suggest that immune maturation and previous HPV exposure may enhance vaccine-induced responses. This age-related enhancement may be attributed to immunological priming effects and mature immune system responsiveness.

### 6.3 Analysis of neutralizing antibody levels at different time points in nonavalent HPV vaccines

**Figure 2:**
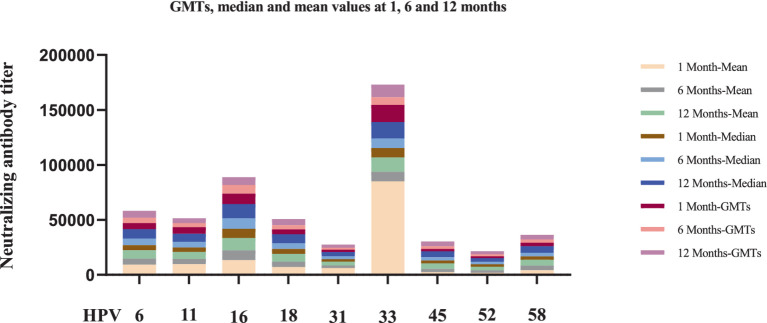
Longitudinal analysis of neutralizing antibody levels against nine HPV subtypes at 1-, 6-, and 12-month after nonavalent HPV vaccination. 8, 20, and 24 recipients tested neutralizing antibody levels in the 1-month, 6-month, and 12-month after nonavalent vaccination, respectively. Each bar chart shows the titers of neutralizing antibodies to each type of HPV at each time point in 9-valent vaccines.

The nonavalent HPV vaccine effectively induced neutralizing antibodies against multiple HPV subtypes while maintaining superior antibody titers across all targeted types (Table 3). Longitudinal analysis of neutralizing antibody responses following nonavalent HPV vaccination revealed variability across HPV types and timepoints (Table 3). At 1-month post-vaccination, GMTs ranged from 1,646 (HPV52) to 15,696 (HPV33). HPV16 and HPV33 maintained the highest absolute antibody levels throughout the study (GMT = 9,545 and 15,696, respectively; Figure 2).

**Table 3:** Analysis of neutralizing antibody levels in 52 nonavalent HPV vaccine recipients at multiple timepoints.

Neutralizing antibody	1-month		6-month		12-month	
	Mean (Median)	GMT	Mean (Median)	GMT	Mean (Median)	GMT
Anti-HPV6	9,204 (4,750)	5,493	5,466 (5,950)	4,715	7,652 (8,600)	6,359
Anti-HPV11	9,714 (4,000)	6,026	4,626 (5,000)	3,580	6,539 (7,500)	4,638
Anti-HPV16	13,486 (8,250)	9,545	8,737 (9,500)	7,740	11,388 (13,000)	7,181
Anti-HPV18	7,132 (4,450)	4,583	4,697 (5,150)	3,767	7,253 (8,200)	5,425
Anti-HPV31	6,284 (2,200)	2082	2,329 (2,580)	1857	3,486 (3,900)	2,864
Anti-HPV33	84,978 (8,550)	15,696	8,669 (8,650)	6,944	13,129 (15,000)	11,480
Anti-HPV45	2,440 (2,700)	2,152	3,024 (3,000)	2,432	4,906 (5,400)	4,271
Anti-HPV52	1,988 (2,100)	1,646	2,158 (2,200)	1,867	3,249 (3,600)	2,773
Anti-HPV58	4,272 (3075)	3,184	4,167 (3,300)	2,987	5,260 (6,000)	4,195

8, 20, and 24 recipients tested neutralizing antibody levels in the 1-, 6-, and 12-month after nonavalent vaccination, respectively.

**Figure 3:**
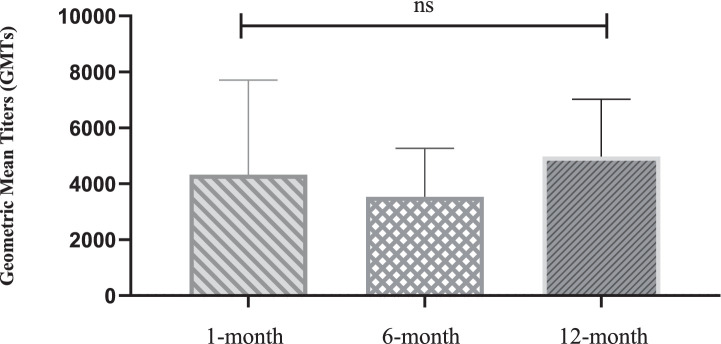
Statistical comparison of geometric mean titers of neutralizing Antibodies at 1-, 6-, and 12-month after nonavalent HPV vaccination. 8, 20, and 24 recipients tested neutralizing antibody levels in the 1-month, 6-month, and 12-month after nonavalent vaccination, respectively. Data presented as geometric mean titers with 95% confidence intervals. Statistical analysis performed using Kruskal-Wallis test. *p < 0.05, **p < 0.01, ***p < 0.001, ****p < 0.0001; ns = not significant (*p* > 0.05).

Notably, several HPV subtypes exhibited non-canonical patterns. For instance, HPV45 showed delayed immunogenicity, with GMTs progressively increasing from 1 to 12 months, whereas HPV52 persisted at low levels (GMT = 2,773 at 12 months). The median-to-mean ratios remained stable for most HPV types, except HPV33, which exhibited marked right-skewing at the 1-month timepoint, indicating subpopulations with exceptionally high responses. No statistically significant differences in neutralizing antibody levels were observed across the nine HPV subtypes at different timepoints after nonavalent vaccination (*p* > 0.05, Figure 3).

### 6.4 Multivariate analysis of HPV16 and HPV18 neutralizing antibodies

To control for potential confounding variables, multivariate linear regression analyses were conducted for HPV16 and HPV18 neutralizing antibodies using log-transformed titers (Table 4).

**Table 4:** Univariate and multivariate analysis of factors associated with HPV16 and HPV18 neutralizing antibody levels.

Variable	HPV16		HPV18	
	Univariate	Multivariate	Univariate	Multivariate
	*p*-value	*p*-value	*p*-value	*p*-value
Vaccine type	0.007	0.089	0.000	0.156
Age (years)	0.000	<0.001	0.000	<0.001
		β = 0.045 (0.028–0.062)		β = 0.038 (0.021–0.055)
BMI	0.830	0.743	0.687	0.892
Temperature	0.565	0.821	0.540	0.736
Sampling months	0.550	0.634	0.255	0.287
Multivariate model statistics	HPV16: R^2^ = 0.387; *p <* 0.001			
	HPV18: R^2^ = 0.341; *p* < 0.001			

β coefficients with 95% confidence intervals shown for significant predictors. Log-transformed antibody titers used as dependent variables. *n* = 84 participants. 12-month bivalent vaccine data excluded due to small sample size (*n* = 6) and biologically implausible values.

In univariate analyses, both vaccine type and age showed significant associations with neutralizing antibody levels (both *p <* 0.05). However, after multivariate adjustment including age, BMI, time to sampling, allergy history, and health status, age emerged as the predominant predictor for both HPV16 (β = 0.045, 95%CI: 0.028–0.062, *p <* 0.001) and HPV18 (β = 0.038, 95%CI: 0.021–0.055, *p <* 0.001) antibody levels.

For HPV16, vaccine type showed a trend toward significance (*p* = 0.089) after adjustment, while for HPV18, vaccine type effects were attenuated (*p* = 0.156). Other covariates including BMI, time to sampling, allergy history, and health status showed no significant associations (all *p* > 0.05).

These findings suggest that age-related factors, potentially including immune system maturation and previous HPV exposure, may be important determinants of vaccine-induced neutralizing antibody responses. The attenuation of vaccine type effects after age adjustment indicates that demographic factors should be considered when interpreting comparative vaccine immunogenicity data.

## 7 Discussion

In this study, serum samples were collected from 84 individuals who had received different types of HPV vaccines: 52 recipients of the nonavalent HPV vaccine, 22 recipients of the quadrivalent HPV vaccine, and 10 recipients of the bivalent HPV vaccine. The consistent completion of full vaccination series across all participants ensures reliable comparative immunogenicity analysis. All dosing intervals adhered to manufacture guidelines, supporting the validity of our vaccine group comparisons. Neutralizing antibody titers against HPV were successfully detected in these serum samples using the pseudovirus-based neutralization assay (PBNA). A titer of less than 1:40 was considered negative, indicating the absence of detectable neutralizing antibodies against HPV in those vaccine recipients. The use of PBNA in this study provide several advantages over other serological methods: it detects all functional neutralizing antibodies in serum samples, offers high reproducibility across different HPV types, and provides direct evidence of protective immune responses rather than surrogate markers.

The bivalent HPV vaccine elicited significantly higher neutralizing antibody titers against HPV16 and HPV18 compared to the nonavalent and quadrivalent vaccines (*p* < 0.001). However, the bivalent vaccine targets only these two subtypes, limiting its coverage. Consistent with previous studies, our findings also demonstrate lower GMTs against HPV18 compared to HPV16 in recipients of the bivalent vaccine (23, 24). The excellent immuno-persistence of the bivalent vaccine provides a specific protection option for high-risk individuals, such as those at elevated risk for HPV16-and HPV18-associated cervical cancer. As detailed in Table 1, recipients of the bivalent vaccine exhibited significantly higher neutralizing antibody levels against HPV16 and HPV18 at the 6-month timepoints (*p* < 0.001). Long-term data were excluded due to small sample size and measurement artifacts.

Our findings are supported by Christine Conageski et al., who demonstrated that the bivalent HPV vaccine possesses strong immunogenicity and efficacy (25). Similarly, Zhu et al. attributed this enhanced immune response to the adjuvant used in the bivalent vaccine (26, 27). Studies have further confirmed the excellent efficacy of the bivalent vaccine in preventing HPV16-and HPV18-associated cervical cancers (28), although it’s limited subtype coverage remains a drawback.

The quadrivalent vaccine showed good stability in inducing neutralizing antibodies but elicited significantly lower antibody titers compared to the other HPV vaccines, suggesting relatively weaker protective efficacy (29). In contrast, the nonavalent vaccine demonstrated significant antibody persistence across extended subtypes, consistent with the findings of Kjaer et al. (30), who reported that the nonavalent vaccine offers the broadest protection against multiple HPV subtypes. Additionally, studies have shown that the nonavalent vaccine is highly effective in inducing immune responses against high-risk HPV types (HPV31, 33, 45, 52, and 58) and in preventing infections with these subtypes. The differential immunogenicity patterns observed among the three vaccine types have important clinical implications. While the bivalent vaccine offers optimal protection against the most oncogenic types (HPV16/18), the nonavalent vaccine provides broader protection against additional high-risk types, representing a transition from targeted to comprehensive HPV prevention strategies.

Mechanistic Basis for Differential Vaccine Immunogenicity. The observed differences in neutralizing antibody induction among HPV vaccines can be explained by several interconnected factors:

First, adjuvant systems play a crucial role in shaping immune responses. The bivalent vaccine employs the AS04 adjuvant system, which combines aluminum salt with monophosphoryl lipid A (MPL: 3-0-desacy 1–4′-monophosphory lipid A). This immune responses and enhances memory B-cell formation compared to the aluminum-only adjuvants used in quadrivalent and nonavalent vaccines (31, 32). Studies have demonstrated that AS40 adjuvant induces stronger and more durable antibody responses through enhanced dendritic cell activation and CD4 + T-cell priming (33, 34).

Second, antigenic competition in multivalent vaccines represents a fundamental immunological challenge. As vaccine valences increases, the immune system must allocate responses among multiple antigens, potentially leading to reduced responses against individual types (35). Higher valences vaccines may experience immune interference between different VLP antigens, with limited antigen-presenting cell capacity and B-cell competition for T-helper cell assistance (36). Previous studies on pneumococcal and meningococcal vaccines have demonstrated similar immunological interference patterns (37), providing a mechanistic basis for the trade-off between breadth of coverage and immunogenicity intensity observed in our comparative analysis.

Third, Differences in VLP production systems and antigen concentrations may also contribute to observed immunogenicity variations. The bivalent vaccine contains higher antigen doses per HPV type (20 μg each for HPV16/18 L1 protein) (38), while multivalent vaccines distribute antigenic load across multiple types, potentially reducing the stimulus strength for any individual type. Additionally, VLP conformational stability and neutralizing epitope presentation can vary between manufacturing processes (39), potentially affecting the magnitude of neutralizing antibody responses across different vaccine platforms.

As shown in Table 2, we observed that the nonavalent HPV vaccine elicited superior immunogenicity in individuals aged >26 years (*p* < 0.0001). This finding is consistent with the results of Huakun Lv et al. (40), who demonstrated that antibody responses in Chinese women aged 27–45 years vaccinated with the nonavalent HPV vaccine were not inferior to those in younger women. The superior immunogenicity observed in older recipients may reflect several biological: (1) rather than reflecting immunosenescence, this may indicate immune system maturation leading to more robust antibody responses. (2) potential immunological priming from prior HPV exposure, older individuals demonstrate more robust secondary immune responses due to prior subclinical HPV exposures or enhanced immune system priming (41). And (3) different baseline immune profiles between age groups. This finding has important implications for vaccination strategies and supports extended age recommendations for HPV vaccination.

As illustrated in Table 3; Figures 2, 3, the nonavalent HPV vaccine effectively induced neutralizing antibodies against multiple HPV subtypes while maintaining superior antibody titers across all targeted types. Among the HPV subtypes, several exhibited non-classical patterns: HPV16 and HPV33 maintained the highest absolute antibody levels (GMT) throughout the study period, HPV45 showed delayed immunogenicity, and HPV52 consistently exhibited low titers. The median-to-mean ratios of most HPV types remained stable; however, HPV33 demonstrated a clear right-skew at the 1-month timepoint, suggesting that subpopulations had unusually high responses. The exceptional anti-HPV33 titers align with structural studies indicating that its L1 protein contains highly immunogenic epitopes (42). The structural homology between HPV L1 proteins may contribute to cross-reactive antibody responses observed in our study (43). Phylogenetic analysis has revealed that closely related high-risk HPV types (such as HPV31/33/45/52/58) share conserved neutralizing epitopes (44), potentially leading to immune boosting effects between related types. This cross-reactivity phenomenon has been documented in both natural infection and vaccination studies (45, 46), and may influence the interpretation of type-specific neutralization patterns in multivalent vaccine recipients.

These findings highlight the inherent trade-offs in vaccine design: broader protection spectrum versus optimized type-specific immunity. Understanding these mechanisms is crucial for future vaccine development and clinical decision-making.

Age-Related Immunogenicity Patterns and Confounding Considerations (Table 4).

Our multivariate analysis revealed that age is a predominant predictor of neutralizing antibody responses, with vaccine type effects being attenuated after demographic adjustment. This finding has several important implications for vaccine immunogenicity assessment and clinical interpretation.

First, the age-related enhancement in antibody responses may reflect immunological priming from subclinical HPV exposures in older individuals, leading to enhanced secondary immune responses upon vaccination (41). Alternatively, immune system maturation may contribute to more robust antibody production in older recipients, contradicting traditional assumptions about age-related immunosenescence in vaccine responses.

Second, the attenuation of vaccine type differences after age adjustment suggests that demographic factors may partially explain the apparent emphasizes the importance of age-stratified analysis and demographic matching in vaccine comparison studies. The differential age effects on HPV16 versus HPV18 responses (stronger age association for HPV16) may indicate type-specific immunological patterns or varying baseline exposure rates to different HPV types in the population.

Third, these findings highlight the complex interplay between host factors and vaccine characteristics in determining immunogenicity outcomes. While our univariate comparisons provide valuable insights into vaccine performance under real-world conditions, the multivariate results underscore the need for careful consideration of demographic factors in vaccine evaluation studies.

The persistence of age as the strongest predictor across both HPV types suggests the chronological age may serve as a surrogate marker for cumulative immune experience and maturation, which could be incorporated into future vaccination strategies and immunogenicity predictions.

The following comparative analyses represent univariate assessments under real-world vaccination condition. Multivariate analysis accounting for demographic factors is presented in Section 5.4.

Currently, the pseudovirus-based neutralization assay (PBNA) is considered the gold standard for the quantitative detection of neutralizing antibodies against multiple HPV types *in vitro* (21). PBNA detects all functional neutralizing antibodies in blood samples, providing an unbiased assessment of antibody levels, independent of vaccine type.

In addition to PBNA, common serological assays used in clinical trials to measure HPV antibodies include Enzyme-Linked Immunosorbent Assay (ELISA) and Competitive Luminex Immunoassay (47–49). ELISA measures total binding antibodies but cannot differentiate between functional neutralizing antibodies and non-neutralizing antibodies, limiting its ability to directly reflect the protective immune effect induced by the vaccine (50). In contrast, PBNA directly detects neutralizing antibodies, making it more reliable for evaluating vaccine efficacy and long-term immune responses.

The Competitive Luminex Immunoassay estimates neutralizing antibody levels by assessing the competition between serum antibodies and labeled, HPV epitope-specific monoclonal antibodies for binding to specific epitopes on HPV virus-like particles (VLPs) (51). However, PBNA uniquely detects neutralizing epitopes that may not be identified by Luminex immunoassays (52–54).

Advancements in PBNA technology, including automated assays based on Gaussia luciferase and three-color PBNA, have significantly improved the throughput of these assays (55, 56). A recent study introduced an adaptive three-color PBNA method that utilizes three fluorescent protein genes, enabling the simultaneous quantification of neutralizing antibodies against three different HPV types. This innovation offers new possibilities for assessing the immunogenicity of highly multivalent vaccines (57).

However, several limitations should be considered when interpreting our findings. First, an important limitation of our study is the absence of baseline HPV DNA screening and prior infection history assessment. Pre-existing natural HPV infection could influence post-vaccination neutralizing antibody levels through immune memory effects, potentially confounding our comparative analysis between vaccine types. Future studies should incorporate comprehensive HPV genotyping and baseline serology to distinguish vaccine-induced from naturally-acquired immune responses. Secondly, this study exclusively utilized the PBNA method without comparison to other neutralizing antibody assays, limiting the ability to cross-validate our results using different methodologies. Third, an important limitation of our study concerns the temporal analysis of bivalent vaccine immunogenicity. The small number of participants completing 12-month follow-up (*n* = 6) and the presence of biologically implausible antibody values necessitated exclusion of these data. This highlights the challenge of conduction long-term immunogenicity studies and emphasizes the need for larger, well-controlled cohort studies to establish reliable persistence profiles for different HPV vaccine formulations. In addition, we have limitations regarding the statistical aspects of the data. To address potential confounding effects, we conducted multivariate analyses for HPV16 and HPV18 neutralizing antibodies, controlling for age, BMI, time to sampling, allergy history, and health status. These analyses revealed that age is the predominant predictor of antibody responses, with vaccine type effects being attenuated after adjustment. This finding adds important nuance to our comparative conclusions and highlights the critical role of demographic factors in vaccine immunogenicity assessment. However, we acknowledge that prior HPV exposure status was not assessed, which represents a limitation that could influence the interpretation of age-related effects. Future studies should incorporate multivariate analytical approaches to control for these factors and provide more robust comparative assessments. Despite this limitation, our study provides valuable preliminary insights into HPV vaccine-induced neutralizing antibody responses, forming a critical foundation for future larger-scale research.

Additionally, the varying and limited follow-up durations of the HPV vaccines complicate the interpretation of long-term immunogenicity and antibody persistence. Larger, longitudinal studies with extended follow-up durations are required to confirm our findings and to assess whether PBNA is suitable as a routine experimental method for detecting neutralizing antibodies.

## 8 Conclusion

HPV vaccines are highly effective in stimulating the production of neutralizing antibodies across diverse populations. Specifically, the nonavalent vaccine is currently the only vaccine that covers seven high-risk HPV subtypes (HPV16, 18, 31, 33, 45, 52, and 58). Its significant protective effect against extended subtypes represents a major advancement in vaccine research and development, marking a transition from subtype-specific protection to broader-spectrum protection. Importantly, our data demonstrate that nonavalent HPV vaccination remains highly effective and may be even more immunogenic in older adults, supporting current recommendations for extended age vaccination programs.

In contrast, the bivalent HPV vaccine demonstrated superior efficacy in generating neutralizing antibodies against HPV16 and HPV18. This suggests that the bivalent vaccine may remain an important option for specific populations, particularly in regions with limited resources and low rates of extended subtype infections.

The mechanistic insights from this study contribute to our understanding of HPV vaccine immunology and may inform future vaccine optimization strategies.

The PBNA method proved to be an effective tool for detecting neutralizing antibodies across various HPV vaccines, offering a reliable approach for evaluating vaccine-induced immune responses. This study provides valuable insights into the immune responses elicited by HPV vaccination and offers guidance for future vaccine development and clinical evaluations.

## Data Availability

The original contributions presented in the study are included in the article/supplementary material, further inquiries can be directed to the corresponding author.
